# Digital technology in national curricula: a dialogue among Iberoamerican countries

**DOI:** 10.3389/fspor.2024.1355967

**Published:** 2024-05-02

**Authors:** Allyson Carvalho de Araújo, Kesia da Silva Xavier, Irene Moya-Mata, Alan Queiroz da Costa, Edwin Alexander Canon-Buitrago, Braulio Nogueira de Oliveira, Ricardo Souza de Carvalho

**Affiliations:** ^1^ Department of Physical Education, Federal University of Rio Grande do Norte, Natal, Brazil; ^2^ Department of Didactics of Physical, Artistic and Music Education, Faculty of Teacher Training, Valencia University, Valencia, Spain; ^3^ Higher School of Physical Education, University of Pernambuco, Recife, Brazil; ^4^ Higher Institute of Physical Education, Universidad de la República, Paysandú, Uruguay; ^5^ Department of Education, Federal Institute of Education, Science and Technology of Ceará, Morada Nova, Brazil; ^6^ Physical Activity Sciences Department, Universidad Católica del Maule, Talca, Chile

**Keywords:** educational policies, Physical Education, curriculum, Media Education, Ibero-American

## Abstract

**Introduction:**

In today's world, digital technologies have become pervasive, impacting every aspect of our lives. Even in subjects such as Health and Physical Education (HPE), which traditionally emphasizes experiential, active, and corporeal learning, there is a growing interest in the role and influence of new technologies. These technologies not only have the potential to transform human movement and health cultures, but they also offer valuable tools to facilitate teachers' work and enhance student learning. Considering the context of the Research Network on Interactive Digital Didactic Materials, which includes researchers from Iberoamerican countries, this research examines how media and digital technologies are considered in the official Physical Education and curriculum documents from Brazil, Uruguay, Chile, Colombia, and Spain.

**Methods:**

The research comprises a content analysis of official National Curricular Proposals of the selected countries. Considering the specificities of each country to publish their curricula policies, we selected eight different documents from five countries (Brazil, Uruguay, Spain, Colombia, and Chile). We looked for indicators expressed by terms such as “media,” “digital culture,” and “technology” in the documents, all of them related to Physical Education.

**Results:**

The findings show arguments and proposals for using technology in Physical Education in all documents. However, the curricula trigger this theme in different forms, as an autonomous or transversal character integrated into subjects such as HPE. This fact may be highlighted by the goals of learning areas, which sometimes trigger teaching themes through technology.

**Discussion:**

Under the Media-Education theory lens, we argue that there is no standard for educational investment in curricula in media and technology. Some documents point to the technology use dimension, while others point to the critical or productive dimension that technology makes possible. The literature highlights the need for an organic approach between these dimensions, and educators and policymakers are asked to rethink their curriculum proposals.

## 1 Introduction

Digital technologies impact various aspects of our lives, including formal education. Even in subjects such as Health and Physical Education (HPE), which traditionally emphasizes experiential, active, and physical learning, there is a growing interest in the role and influence of new digital technologies. This debate permeates their potential to transform human movement and health cultures, facilitate teachers' work, and enhance student learning (1), and, to a lesser extent, issues such as data surveillance (2).

In the context of Ibero-American countries, it is possible to observe pedagogical experiences that already incorporate themes such as media and technologies related to HPE (3, 4). However, investigating how curricular policies develop this topic is a timely advance, allowing not only an overview of local demands but also an educational contextualization of these countries to other global scenarios. Furthermore, this type of research identifies the weaknesses and strengths of curricular proposals and establishes the basis for more consistent collaboration in producing new versions adapted to the specific reality of each country.

Some Latin-American curriculum analyses focusing on the media theme have already been carried out (5, 6). In the case of Brazil, Araújo et al. (6) identified a growing interest in media and technology, emphasizing critical thinking about media narratives and not about the production of media content. In this sense, the authors highlight the need to broaden the perspective on digital culture.

In Uruguay, several studies of a historical nature (7), sports practices in training (8), and the discourses of Physical Education in the country's official curricular programs (9, 10) have contributed to thinking about the construction of the body using different types of biopolitical, media or pedagogical technologies.

On the other hand, in Colombia, authors such as Quilindo (11), Arcila, Canchala, and Granados (12), and Hurtado and Molina (13) focused on understanding curricular guidelines within the scope of Physical Education linked to interculturality, competence, and pedagogical practices.

In Spain, current educational legislation promotes the development of digital competence among teachers and students to combat gender disparity (14). However, studies such as that by Menescardi et al. (15) demonstrate that the applications used by teachers are currently scarce and generic, leaving aside the use of different technological tools on the market.

Although there are similarities in the field of education, Ibero-American countries present significant differences in their understanding of Physical Education. In a comparative study between Brazil and Chile, Barcelos, Almeida, and Doña (16) identified some similarities and differences that lead us to think, for example, that Physical Education is configured as a curricular component in Brazil, while it is considered a mere activity in teaching in Chilean schools.

Even though there is some systematization in the cases mentioned above, except for the Brazilian case, there is no research focus on digital technologies or articulation between advances and setbacks between the different Ibero-American countries.

For the HPE area, it is imperative to think about technology issues since one of the main issues in media consumption by young people (10–15 years old) is body dissatisfaction (17). In this sense, interventions in media literacy can reduce such discontent (17). A study conducted in Spanish universities about media consumption by young people identified that social media is the primary source of information and, simultaneously, the least reliable source, leading to the dissemination of fake news (18). Thus, there is a lack of media literacy from a critical perspective, even among university students (18).

In a way, teaching Physical Education through media or technology, with pedagogical intention, has become an increasingly recurrent topic of study (19), but no study encompasses Ibero-American countries. The challenge of producing more collaborative, multidisciplinary models and being open to using multiple languages remains (20), as does the challenge of using digital educational resources in schools (21). Particularly in the context of Ibero-American countries, the absence of a comparative study prevents the development of more consistent educational policies aimed at their appropriation by the school community.

Based on this context, this article aims to analyze how digital media and technologies are considered in official Physical Education and curricular documents in Brazil, Chile, Colombia, Spain, and Uruguay. The idea is collaborating with knowledge production on this theme in the Ibero-American context.

### 1.1 Media, technology, and curriculum

The previously presented context reveals that emerging social elements like technology can and do change realities. For example, digital technology has become part of educational contexts and environments, which have had to deal with new needs and demands that did not exist before.

Nowadays, many devices that present several media narratives (e.g., TV, newspapers, magazines, and, especially, the Internet) compete with other elements in the traditional school reality, such as teachers and their blackboards and textbooks. In a cultural moment when the student generation has grown up with digital media as an ongoing tool to be informed, the centrality of school has been questioned. As per Prensky (22), both educators and students find themselves constrained by a curriculum that, due to historical reasons, struggles to keep pace with the swift advancements and influence of technology.

On the one hand, students are connected all the time, given that 74% of them claim to have mobile phones (23) with several purposes, including exchanging messages, watching videos, listening to music, etc. On the other hand, teachers are still adapting to these forms of information access and face pedagogical challenges regarding how to deal with this new situation. According to a more robust Brazilian report (24), 52% of Brazilian teachers reported learning to use computers and the Internet by taking courses and seeking updates.

In encountering these two traditional characters in the school space (teachers and students), there is an agenda of what should be taught—the school curriculum. More than being a prescriptive text, the curriculum should be understood to articulate the aspirations of public policies, pedagogical theories, and school practices. Therefore, the school curriculum is a contested space, a debate field around what should be taught and learned as knowledge considered legitimate to be addressed in schools (25).

With the popularization of digital technologies and their recognized educational potential, the anachronism of school curricula and their standardized knowledge concerning people's lives outside the school walls intensifies (26). This fact highlights the difficulty in developing a curriculum that accommodates the impact of digital technologies.

In a study of six European countries, Bruni (27) describes the difficulty when media and technology became an independent learning area. The strategy of becoming technology as an independent area created disciplinary boundaries by focusing on understanding technology, regardless of the demand to relate it to the subjects of the thematic universe of students (27).

Passarelli and Azevedo (28) highlight two “waves” in society that mark governmental concerns through policies and programs for digital inclusion and different forms of knowledge appropriation and production on the Web. According to the authors, the first wave can be defined between 1995 and 2005, when attention focused on access and the provision of technological infrastructure through public policies for digital accessibility to realize citizenship. From that period onwards, they began to perceive and analyze social, technical, and sociotechnical realities, bringing new approaches to meet the increasing appropriation of new technologies and constructing new identities and narratives, thus characterizing an ongoing process they termed the second wave (28).

According to those data, the educational policies and pedagogical practices initially focused on the dynamics of using technologies (29). Moreover, for a critical, actively engaged perspective of technology in education, it is necessary to explore other nuances beyond the technical use of technological devices. In this sense, Rivoltella (30) and Fantin (31) point out the possibility of understanding education's involvement with media and technology through the three dimensions of the Media Education theory: the methodological, the critical, and the productive.

The first dimension focuses on the methodological or technological context. In this dimension, media education is conceived as making education with media and relies on an instrumentalist view of media within the scope of didactic methodology. From this perspective, media education is considered a resource for education to enhance didactics by teaching with other means, aiming to overcome the traditional scheme and replace textbook support with cinema, computers, and TV programs, among others (31). Media then serves as a resource in an instrumental pedagogy, herein named “Educate with media and digital technology.”

The second dimension focuses on the critical context. In this dimension, media education provides education about the media as an object of study in which understanding, interpreting, and evaluating the content of various media. Sometimes, this perspective may fit into a moral pedagogy, which, through the critical ideological reading of social sciences, aims to defend users, cultivate their criticism, and make individuals reflective (31). This dimension is called here “Educate about digital media and technology.”

The third dimension focuses on the productive context. In this dimension, media education is understood as making education through media or within media involving professional training. Conducting media education means using media as a language, a form of expression and production. As nobody learns how to read without learning how to write, media education is not done solely through critical reading and instrumental use of media; it is necessary to learn to “write” in the media languages. This perspective fits into a functional pedagogy, aiming at the interaction of individuals with media and promoting creative and critical knowledge of their languages (31). This dimension is named “Educate through media and digital technology.”

In the context of Physical Education, many teachers still need to develop a critical media pedagogy focused on changes in the physical culture. In contemporary literature, only two studies on Iberoamerican curricula highlight the fragility of the relationship between technology and the Physical Education curriculum (5, 6). Therefore, it is necessary to put research effort into how the official curriculum documents understand the relationships between technology and Physical Education as a strategy and then explore how Iberoamerican educational policies are organized based on social demands and contemporary pedagogical practices.

## 2 Method

To conduct this study, we conducted a multiple case study (32) through a comprehensive content analysis (31) of curricular documents and official curriculum policies from Brazil, Uruguay, Chile, Spain, and Colombia. Our choice to use a multiple case study was deliberate, respecting the distinct national contexts of each country. This method enables the investigation of varied outcomes arising from contextual differences and facilitates the identification of common findings across the studies. Multiple case studies have been used to compare conceptualizations and positions of technology education in contemporary literature (34, 35).

We focused on curriculum documents from Brazil, Uruguay, Chile, Spain, and Colombia due to the authors' nationalities (or their residence/work experience exceeding five years) and intimate knowledge of their respective national curricula. Their profound understanding of the local educational landscape, encompassing shifts in curriculum policies and language, provided the foundation to pursue deeper comparisons among the documents.

### 2.1 Documents

The study sample encompassed the current curriculum documents of the selected countries. Considering the specificities of each country to publish their curricula policies, we selected eight different documents from five countries (Brazil, Uruguay, Spain, Colombia, and Chile) in the Iberoamerican region (see Table 1).

**Table 1:** Chart showing an overview of technology and media in the physical education curriculum documents of Iberoamerican countries.

Country	Document	Publication year	Learning area	Specific technology learning area	Term to technology debate
Brazil	Base Nacional Comum Curricular	2018	Educação Física (Physical Education)	No	Digital information and communication technologies
Uruguay	Marco Curricular Nacional	2022	Educación Física (Physical Education)	Comunicación y sociedad; Ciencias de la computación (optional)	Technology
	Plan de Estudios Educación Básica Integrada	2022			
Spain	Ley Orgánica 3/2020	2020	Educación Física (Physical Education)	Tecnología y Digitalización; Ciencias y Tecnología (optional)	Information and communication technologies
	Real Decreto 157/2022	2022			
	Real Decreto 217/2022	2022			
Colombia	Lineamientos curriculares para la educación física, recreación y deporte	2000	Educación Física (Physical Education)	No	Technology
Chile	Bases curriculares educación física y salud 1° Básico a 2° Médio	2016	Educación Física (Physical Education)	Tecnología	Information and communication technologies

### 2.2 Data collection and analysis procedures

Following Bardin's (33) model, content analysis unfolded into three sequential stages, namely: (1) Preliminary analysis, (2) Exploration of the material, and (3) Processing of results, inference, and interpretation. Initially, we thoroughly read the documents to understand their structure and identify media and technology themes to pinpoint text units for analysis. Following the methodology, a text unit could be a word, a theme, or an expression linked to a specific topic, which will serve as a guiding principle for coding in the subsequent analysis stage.

In the second stage, in our case, we decided to use three words as text units: “media,” “digital culture,” and “technology”. We also aggregated these text units with some related expressions, such as “communication devices” and “digital competence.” The reason to consider these expressions is the ramifications on Portuguese and Spanish languages to refer to some terms. While exploring the material analysis stage, text units underwent initial quantitative analysis to ascertain their frequency and distribution on the countries' documents. At this moment, we can assess the representativeness of each theme within the documents and isolate them to better understand their significance.

**Figure 1:**
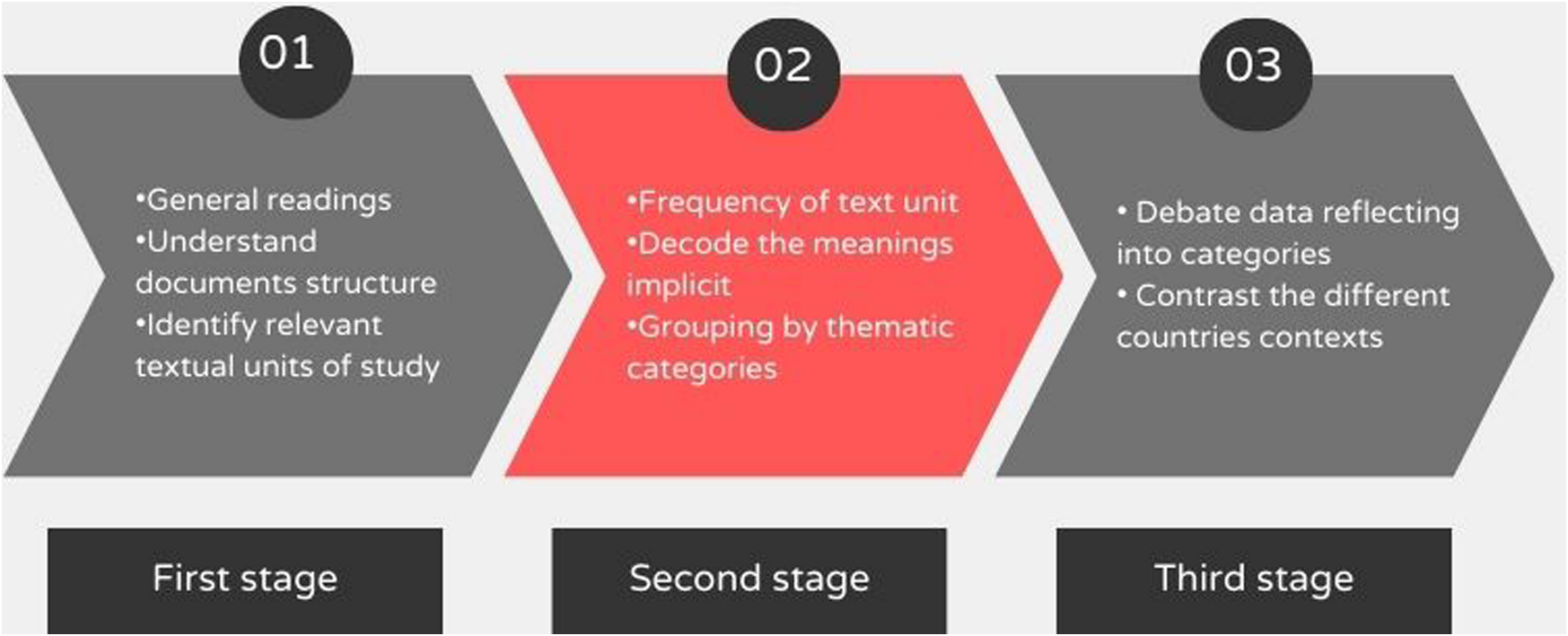
Analysis procedures.

Subsequently, a qualitative exploration was conducted to contextualize their significance within the document. We excerpted the usage contexts of each text unit appearing in the documents and analyzed the textual meanings to examine and decode implicit meanings for their categorization. We used the three dimensions of the Media Education theory—methodological, critical, and productive (30, 31)—as a theoretical framework encompassing the meanings derived from the data collected from the documents, thereby enabling the organization of analysis categories (30, 31). In the third stage, we reflected on the presence, absence, and inconsistencies concerning media and technology in these documents, contrasting the contexts of diverse international educational systems. Figure 1 depicts the methodological procedures.

## 3 Results and discussion

The initial examination of the curriculum documents unveiled three primary observations: (i) the recognition of the importance of digital technologies, (ii) the similarity in nomenclature use, and (iii) digital technologies guiding some approaches to Physical Education content in the documents.

Two points can be observed from the high frequency of terms appearing in the data collection. Even the oldest curricula documents, such as “Lineamientos curriculares para la educación física, recreación y deporte” from Colombia (36), highlight the importance of technologies in education, including Physical Education.

Different expressions in all documents show us how curriculum policies are committed to defending the use of technology in school education, as it is possible to see in the following examples (free translations into English):

Skill development is promoted through problem-solving situations, collaborative proposals, prototypes, research, projects, case studies, games, artistic activities, and didactic outings, incorporating technology in its potential for learning and its value as an object of study (37).

or

[…] given the intrinsic relationship between youth cultures and digital culture, it is essential to expand and deepen the learning constructed in previous stages (38).

The justification for incorporating media and technology in schools covers several aspects. The two main aspects highlighted are (i) the importance of integrating media and technology in education and (ii) the need to cultivate digital skills in line with social transformations.

It is worth mentioning that the term “technology” stands out as the most frequently employed term in the documents (refer to Table 1). This term is sometimes used independently, as we observed in the documents from Uruguay (37, 39) and Colombia (36). However, in other instances, the term “technology” is linked to more widely recognized expressions, such as “Information and communication technologies,” as evident in the documents from Spain (40, 41) and Chile (42). Furthermore, it is explicitly combined with the notion of digital as seen in the expression “Digital information and communication technologies” in the document from Brazil (38).

The frequency of word usage indicates that the most frequently mentioned words might signify the primary concerns within a document. While a mere count doesn't directly infer meaning, understanding word frequency hints at representativeness and aids in conducting additional analyses (such as cross-sectional or comparative) to detect any fluctuations, inclines, or alterations of these terms in curriculum documents (6).

The use of “Information and communication technologies” or “Digital information and communication technologies” aligns with the concept of Media and Information Literacy as introduced by UNESCO in 2011 (43), which characterizes it as a kind of literacy expected at present. UNESCO categorizes media and information as two forms of literacy due to its correlation with the evolving demands of the contemporary era:

On the one hand, information literacy emphasizes the importance of access to information and the evaluation and ethical use of such information. On the other hand, media literacy emphasizes the ability to understand media functions, evaluate how those functions are performed, and rationally engage with media for self-expression (44).

In our exploration of the eight documents from the five Iberoamerican countries, we identified a total of 204 instances where the themes of “media,” “digital culture,” and “technology” were explicitly addressed in the content associated with the Physical Education curriculum. The documents from Brazil (38) and Uruguay (37, 39) stood out with the highest thematic frequency, accounting for 53 and 51 occurrences, respectively (see Table 2).

**Table 2:** General framework for keyword searches.

#	Document/Keywords	Media	Digital culture	Technology	Total
1	Base Nacional Comum Curricular (Brazil)	20	10	23	53
2	Marco Curricular Nacional and Plan de Estudios Educación Básica Integrada (Uruguay)	29	0	22	51
3	Ley Orgánica 3/2020, Real Decreto 157/2022, and Real Decreto 217/2022 (Spain)	18	13	6	37
4	Lineamientos curriculares para la educación física, recreación y deporte (Colombia);	27	0	5	32
5	Bases curriculares educación física y salud 1° Básico a 2° Médio (Chile)	6	5	20	31
	Total	100	28	76	204

Generally, the media theme exhibited the highest frequency of occurrences, closely followed by the technology theme. These findings align with previous studies (45), which indicated that certain curriculum documents do not specifically address digital culture.

Upon isolating and categorizing each expression, our next step involved analyzing the implicit meanings associated with its use by aligning it with the dimensions or contexts of Media Education theory (30, 31). These dimensions have previously served as the background for examining Physical Education Teacher Education (46) and school curriculum document analyses (5, 6) related to the technology approach.

Despite using the previously mentioned dimensions/contexts of the Media Education Theory, which is intended to encompass all pedagogical intentions related to educational media use, some occurrences did not align with any specific category (see Table 3). This aligns with other studies on Physical Education curricula (6, 45), with the predominant category being about general concepts, explanations, and arguments concerning the significance of media and technology. We argue that the emergence of this theme in the documents is more due to a response to the cultural transformations resulting from the co-evolution of technology and education (47) rather than a proactive approach outlining how to integrate technology into the curriculum.

**Table 3:** Analysis’ category by country.

Analysis category/Country	General concepts and relevance of media and digital technology	Educate with media and digital technology	Educate about digital media and technology	Educate through media and digital technology	Total
Brazil	12	4	24	13	53
Uruguay	30	3	15	3	51
Spain	22	5	9	1	37
Colombia	24	0	8	0	32
Chile	6	25	0	0	31
Total	94	37	56	17	204

Sibília (26) proposes that contemporary schools face a crisis as they grapple with more comprehensive social transformations, particularly those propelled by technology, leading to a certain degree of obsolescence. Based on the general concepts category, we observe whether this scenario might unfold in Iberoamerican curriculum policies, which currently appear to lack justifications for incorporating such elements into their proposals.

Regarding the categories within the Media Education theoretical framework, our analysis reveals a preference in curricula documents for the “Educating for media and technology” category, closely followed by the “Educate with media and digital technology” category. This stands in contrast to the perspectives of Buckingham (29) and Ferreira Jr and Oliveira (5), who argue that most media and technology teaching tends to be instrumental, focusing on developing specific content with no critical positioning. However, these results align with a similar previous study (45), which also identifies the “Educating for media and technology” category as the most representative one.

It is crucial to emphasize that approximately three decades ago, concerns about media narratives as a competing pedagogy with traditional schooling were already raised in the education field (48, 49). At the turn of the century, these concerns gained momentum, with various perspectives asserting that media has a pedagogical influence on how individuals shape their identities in contemporary times (50, 51).

From our perspective, this wave has now permeated curriculum policies, reaching all learning areas, including Physical Education. This field has already begun exploring media narratives related to Physical Education content/themes (e.g., sports, body image, and physical activity) through various media formats such as comics and apps, as evidenced by Souza Júnior et al. (52), Oliveira and Fraga (53), and Díaz-Barbosa et al. (54).

For the last category, “Educating through media and technology,” it is noticeable that it had less than 50% of occurrences than the other two categories. Another remarkable observation within this category is that all occurrences are concentrated in three countries: Brazil, Spain, and Uruguay. Brazil leads with 13 instances, while Colombia and Chile have no occurrences (refer to Table 3).

The observation that the category “Educating through media and technology” remains the least represented dimension in curriculum documents is not a novel discovery, as evidenced by previous studies (6, 45). It is particularly noteworthy that, in various instances, this category is employed in a propositional manner, serving as learning objectives or desirable skills, as illustrated in the following examples (free translations into English):

Evaluate the impact of digital information and communication technologies (ICT) on the formation of individuals and their social practices to critically use this media in the practices of selecting, understanding, and producing discourses in a digital environment (38).

Uses digital media to collaboratively produce and select the appropriate format to present information. Attributes the corresponding authorship when using others' productions with adult mediation. Analyzes and reflects on the validity of digital content and begins to use tools and strategies to identify reliable sources. Identifies ethical implications and risky situations in the use of social networks. Recognizes the construction of their digital footprint and identity and the risks of publishing personal data. Reflects with adult guidance on the time dedicated to the use of digital media and the objectives of such use (39).

The discrepancy in the productive category of the Media Education theory between curricula was a particularly intriguing discovery, especially within the Iberoamerican context. However, such learning objectives continue to challenge educational practices. The challenge is rooted in the implication that teachers and students must be adept at using multimodal narrative constructions with digital technologies, particularly as they increasingly become prosumers (55)—individuals who act as both producers and consumers of content simultaneously.

It is a fact that the assessed Iberoamerican nations share the goal of integrating technologies into their educational systems as outlined in the document “Educational Goals 2021: The Education We Want for the Bicentennial Generation” (56), released by the Organization of Ibero-American States for Education, Science, and Culture (OEI). This document contains an ambitious vision for the curriculum:

A meaningful curriculum is one that connects students' interests with their ways of life, adapts to their learning rhythms […] that regularly incorporates the use of information technologies, includes the development of artistic and sports education in a relevant and balanced manner, and is guided to ensure that all its students are well in school and able to learn (56).

Nevertheless, each country deals with its own challenges, achievements, and goals in building curricula. Conceptualizing curricula as a contested terrain that involves deliberations about what should be taught and learned within the realm of recognized knowledge (25), we understand that curricula should, in essence, mirror the evolution of learning areas within their specific context.

For instance, in the case of Chile, the integration of technology in schools has been established as a national policy since 2005, initially through the Enlaces project (57) and subsequently with the incorporation of information and communication technologies (ICT) standards in initial teacher education (58). While this policy highlights the relevance of technology in education as an integral aspect of modern life, it also underscores the essential role of teachers in mediating the use of technology in educational settings to mitigate potential risks. Consequently, there is an emphasis on limiting the use of technologies to pedagogical tools (59).

Examining the development of knowledge on Physical Education concerning technology in the Chilean context, finding a prevalence of references in the Physical Education curriculum related to general concepts and the category' education with media and digital technology' is unsurprising. The national literature offers examples of the instrumental use of technology, aiming to facilitate or motivate students. This perspective aligns with the observations of Quintero (60), who suggests that technological devices can be utilized in various moments and situations, presenting new possibilities for Physical Education teachers to incorporate ICTs into diverse physical and sports activities. The potential of technological devices to improve and enhance physical activity, creating new forms of motivation, is also acknowledged, positively impacting the teaching and learning process (61). Following this trajectory, Pinilla et al. (62) present various apps that promote the use of ICTs in Physical Education classes and facilitate social media as a mediator.

In the context of Uruguay, it is crucial to clarify that both the Common National Curricular Framework and the presentation of the Physical Education curricular component are relatively new (37, 39). For this reason, their implementation/adaptation to educational systems is currently in progress.

Uruguay exhibits a noticeable concentration of data regarding the category “Educate about digital media and technology,” emphasizing fostering students' critical understanding of media narratives. However, the production of knowledge in Physical Education in Uruguay, particularly those integrating studies with media, is still relatively unexplored. Only the work by Piovani (63) explores the possibilities and limitations of incorporating computers into Physical Education classes, using experiences with blogs to facilitate pedagogical exchange between students from different countries. While this work reflects a productive dimension/context within the Media Education theory, being an isolated production implies that it may not represent a broader intellectual movement that can be reflected in the curriculum.

In contrast, Brazil stands out as a country with a tradition of studies on media and technology related to Physical Education since the 1990s (64). The literature on Physical Education is also grounded in Media Education Theory (65–68), whose dimensions/contexts are recognizable in theoretical debates and applied teaching experiences.

Given this Brazilian background, where Physical Education is considered a research sub-field of media and technologies (69), it is understandable that the two most representative categories are “Educate about digital media and technology” and “Educate through media and digital technology.” The Brazilian curriculum policy has also been investing in understanding Physical Education as a language (38, 70) and exploring various languages to teach prescribed skills (71). Building on this foundation, there are also experiences of continued Physical Education focusing on Media Education Theory (72, 46, 20).

Part of our findings are aligned with previous studies adopting a similar methodology (6, 45) where national curricula propose engaging with various technologies (analog and digital) primarily from a critical perspective as a means to enhance students’ abilities in interpreting media narratives and critically reflecting on Physical Education content.

However, our findings also diverge from some results of previous studies. While Araújo et al. (6, 45) indicate that the second most recurrent category is “Educating through media and technology,” our results reveal that the category “Educating with media and technology” exhibits the second highest recurrence. This implies that countries such as Australia, New Zealand, and Brazil emphasize developing their students' skills in producing media narratives rather than the instrumental use of technology for teaching Physical Education, as observed in the results of our set of Iberoamerican countries. In the documents, no explicit indication may explain the observed difference. Nonetheless, it is crucial to consider the various cultural, historical, and contextual distinctions between developed English-speaking countries and developing countries in Ibero-America, including digital technology access.

In general, we can identify both similarities and differences in the investments related to media and technology within the context of Physical Education across the curricular policies of the analyzed countries. Recognizing the tradition and direction of each context concerning investments in media and technology is a valuable approach to glean insights from the variations found in this group of countries. Despite their shared goal of developing education with media support (56), these countries adopt different strategies in shaping their curricular policies.

## 4 Concluding thoughts

Revising school curricula is a struggle for educational policies, particularly concerning media and technologies, as these challenges become more pronounced with the rapid growth of digital technology in our daily lives. This expansion gives rise to imperative technological narratives within the educational sphere (73), fostering a perception that schools consistently lag social dynamics (26) in the so-called postindustrial or information-age society.

This study focuses on countries with historical and cultural affinities organized around OEI. The findings indicate that the curricular documents of Brazil, Colombia, Chile, Spain, and Uruguay acknowledge the relevance of media and technology in school education, encompassing both a broad perspective and within the realm of Physical Education.

Specifically, our findings show that Ibero-American PE curricula privilege a critical perspective of media and technology, followed by an instrumental use of technology to teach Physical Education.

Ultimately, we acknowledge that documents alone do not change educational realities. In that sense, we admit the study limits are confined to official curriculum document analysis. Nevertheless, we also assert that teachers employ a curriculum document as a foundation for decision-making in teaching, shaping how they interpret and implement content within their specific context. While we recognize that the pedagogical approach is rarely uniform across all school settings, adhering to a documented curriculum, we also affirm that these curricula serve as crucial guidelines for teachers' work. Exploring how Iberoamerican PE teachers use media and technology to teach Physical Education, whether they align with official curricula, could be the next step in this research agenda.

### 4.1 Practical implications

The curriculum should be perceived through a historical lens, depicting the continuous evolution of knowledge essential for forthcoming generations concerning pedagogical practices. Hence, the curriculum policies analyzed hold the potential to instigate change, notably in embracing novel pedagogical approaches and educational settings. Such transformation is integral to a dynamic system, adapting alongside the burgeoning influence and variety of modern technology.

Based on the data and results presented, we understand that the practical application of the reflections proposed here can be measured by listening to physical education teachers from these countries and how they translate the guidelines of official documents into their physical education classes.

Although not the focus of this study, as mentioned in the text, in the case of Brazil, there are already several ongoing experiences with the use of various platforms and digital devices in the three dimensions proposed by Media Education theory (17). Drawing inspiration from these experiences, we can mention the use of podcasts, radio, comic books, photography, printed magazines, cinema, digital games, social media, Internet, television, and print newspapers as support for the knowledge of subjects or themes related to the contents of physical education classes.

The experimentation of different contents and practices contextualized by media language or even from recording students' classes and activities can be an essential and significant collection to discuss their experiences and how these processes are constructed. Less than devices, technology should be understood as a language to connect teachers and students on pedagogical approaches.

Finally, it is worth noting that all these demands that we refer to in the field of Physical Education are perceived as opportunities for physical education teachers to continue seeking ways to implement their practices in an updated, meaningful manner that values other ways of seeing the possibilities that the connected contemporary world can offer.

## Data Availability

The original contributions presented in the study are included in the article/Supplementary Material, further inquiries can be directed to the corresponding author.
